# The Role of Heart-Rate Variability Parameters in Activity Recognition and Energy-Expenditure Estimation Using Wearable Sensors

**DOI:** 10.3390/s17071698

**Published:** 2017-07-24

**Authors:** Heesu Park, Suh-Yeon Dong, Miran Lee, Inchan Youn

**Affiliations:** Center for Bionics, Korea Institute of Science and Technology (KIST), 5, Hwarang-ro 14-gil, Seongbuk-gu, Seoul 02792, Korea; pheesu@kist.re.kr (H.P.); suhyeon.dong@kist.re.kr (S.-Y.D.); miran@kist.re.kr (M.L.)

**Keywords:** HRV parameters, activity recognition, energy expenditure estimation, wearable sensors, mobile healthcare system

## Abstract

Human-activity recognition (HAR) and energy-expenditure (EE) estimation are major functions in the mobile healthcare system. Both functions have been investigated for a long time; however, several challenges remain unsolved, such as the confusion between activities and the recognition of energy-consuming activities involving little or no movement. To solve these problems, we propose a novel approach using an accelerometer and electrocardiogram (ECG). First, we collected a database of six activities (sitting, standing, walking, ascending, resting and running) of 13 voluntary participants. We compared the HAR performances of three models with respect to the input data type (with none, all, or some of the heart-rate variability (HRV) parameters). The best recognition performance was 96.35%, which was obtained with some selected HRV parameters. EE was also estimated for different choices of the input data type (with or without HRV parameters) and the model type (single and activity-specific). The best estimation performance was found in the case of the activity-specific model with HRV parameters. Our findings indicate that the use of human physiological data, obtained by wearable sensors, has a significant impact on both HAR and EE estimation, which are crucial functions in the mobile healthcare system.

## 1. Introduction

As mobile healthcare systems have become widely used, users have begun to expect increasingly accurate performance with better appearance. The appearance of the mobile healthcare system relies highly on the development of sensing devices, either external or wearable. Due to many limitations of external sensing devices, wearable sensors have increasingly attracted the interest of both users and researchers. Recently, many wearable sensors have been developed for many applications, such as medical, sports, and commercial fields (see a recent review in [1]). For researchers investigating the mobile healthcare system, it is also challenging to utilize multi-dimensional information collected from existing wearable sensors for more accurate performance.

To guarantee the performance of the mobile healthcare system, two crucial functions are required: human-activity recognition (HAR) and energy-expenditure (EE) estimation. The former, which is also called the HAR problem, has attracted many researchers since the late ‘90s (see recent reviews in [2,3]). With the development of computing technologies, it becomes possible to recognize human activities, especially ambulatory activities, with significantly high accuracy. Researchers have recently reported very high recognition performance, from 97 to 99%, under different approaches [4,5]. The latter function is also broadly implemented in the mobile healthcare service and is represented as calorie consumption. These two issues are closely related to each other in that the EE estimation is accurate assuming that the activities of the monitored person are properly recognized [6].

The most frequently used sensor in the mobile healthcare system is the tri-axial accelerometer. Single or multiple accelerometers are broadly used for the HAR problem and EE estimation. However, as mentioned in a recent review by Lara and Labrador [3], physiological signals such as heart rate, respiration rate, and electrocardiogram (ECG) have attracted little interest. The specific reason we pay attention to physiological signals is that the information provided by the accelerometer is insufficient for recognition of some confusing activities in terms of acceleration. Furthermore, an accelerometer has a critical drawback in cases of little or no movement but with obvious energy consumption, for example, sedentary work. One previous study has proven that heart-rate variabilities reflect qualitative differences in static and dynamic activities [7].

Biomedical sensors for physiological signals have continuously developed. On the other hand, as reviewed by Liu and Liu, recent biomedical sensors have become wireless, portable, and wearable on the platform of mobile phone. Yet, the method of analyzing the collected physiological signals is still a challenge [8].

Based on these considerations, we expect that such physiological signals may provide us with additional information for better recognition of human activities and prediction of EE, even for such cases. To the best of our knowledge, no study has been performed yet to solve these drawbacks for both issues (HAR and EE estimation) with an approach that exploits human physiological signals.

In this study, we aim to recognize human ambulatory activities and estimate EE using our database composed of accelerometer and physiological signals, collected from 13 voluntarily participating subjects in a laboratory environment. To investigate the role of physiological signals in both issues, we compare the recognition and estimation performances with and without ECG data.

The organization of the paper is as follows. We first give a brief review of some of the existing approaches for HAR and EE estimation using wearable sensors. Then, we introduce our database and the wearable sensors used in this study. Next, we describe our approaches and experimental results of HAR and EE estimation. Finally, we conclude this paper with a discussion.

## 2. Related Work

### 2.1. Activity Recognition

The HAR problem, especially for ambulatory activities, has been widely investigated with tri-axial accelerometers. The use of an accelerometer is accompanied by several issues, such as the number of sensors, attachment locations, and classification methods.

First, in terms of sensor quantity and locations, Bao and Intille used five locations of accelerometers (wrist, ankle, thigh, elbow, and hip) with 84% recognition accuracy but reported high obtrusiveness [9]. Khan et al. used only one accelerometer on the chest with high recognition accuracy of 97.9%, but the attachment location caused moderate obtrusiveness [4]. He and Jin reported 97.51% recognition accuracy with a single tri-axial accelerometer inside a trouser pocket, where it is relatively less obtrusive [10]. The use of a multi-position system can be advantageous in terms of information quantity, but too many sensors can cause discomfort for the user. For these reasons, several studies for HAR using wrist-worn accelerometer is starting to emerge [11,12,13]. A wrist-worn device is more convenient to wear than body-fixed-sensor at the hip or torso and it can be worn continuously during free-living. Moreover, as also mentioned above, it is possible for a single sensor to achieve high accuracy on ambulatory-activity recognition. Thus, we use a single accelerometer on the wrist of the individual’s dominant arm, where it does not obstruct ambulatory activities, and an additional sensor for a physiological signal.

Second, classification methods are diverse but almost always operate in a supervised fashion. For example, decision trees [14], support vector machine (SVM) [10], artificial neural network [4], and ensemble of classifiers [15,16] are used. For the selection of a proper classification method for the mobile system, we need to use a simple method with a small computational load. Otherwise, classification may cause additional discomfort to users due to its computational delay. We will compare the performances of several simple classical classifiers.

In this study, we aim to investigate the effect of a human physiological signal, specifically ECG signal, on HAR and EE estimation and propose a novel paradigm for the mobile healthcare system using wearable sensors. Only a few studies were found on the use of a physiological signal for an additional sensor. Tapia et al. [17] reported that they used a heart-rate monitor with five tri-axial accelerometers, but they could not find any improvement in recognizing ambulatory activities due to the usage of a heart-rate monitor. On the other hand, Centinela, a system proposed by Lara et al., reported that vital signs are useful to discriminate certain activities [15]. Li et al. also proposed a multi-modal approach utilizing ECG and accelerometer as well as a fusion of multimodal and multi-domain subsystems in nine-category physical activity database [18]. Except one study by Tapia et al., activity recognition with the combination of physiological and accelerometer signals outperformed the classification by single modal approach.

### 2.2. Energy Expenditure Estimation

There are several existing methods to measure human physical activities: self-reporting, indirect calorimetry, double-labeled water (DLW), and portable monitors (such as ECG and accelerometers) [19]. Among them, an accelerometer is most widely used, especially for estimation of EE, due to its small size, portability, low power consumption, and low cost [20]. Moreover, Plasqui and Westerterp reviewed several commercialized accelerometers by comparison with the DLW technique, which is known as the gold standard in EE estimation, and found one accelerometer having reasonable correlation with DLW-derived EE [21].

Bouten et al. used a tri-axial accelerometer to estimate EE for both sedentary (sitting, writing, and arm work) and walking (intensity varies from 3 to 7 km/h) activities with correlation coefficients of 0.82 and 0.96, respectively [22]. Puyau et al. validated two commercialized accelerometer-based activity monitors in various activities of children in terms of EE estimation [23]. Wang et al. also used a tri-axial wrist-worn accelerometer for estimation of EE in a large dataset of several activity categories [24].

However, as reported in [21], accelerometer-based EE estimation is not yet a perfect replacement for DLW-based techniques. To improve the performance of EE estimation using an accelerometer, some studies suggested activity-specific models [6,25]. This approach may provide better performance, but users must submit their data for every activity to develop each model. Instead, we will compare single and activity-specific models with an additional ECG sensor capturing physical changes in the human body. Considering the differences in physiological changes during static and dynamic activities [7], we assume two separate models for static and dynamic activities (we call this approach activity-specific), which may help to achieve better estimation.

Some researchers have already used additional physiological signals such as heart rate (HR) for more accurate estimation. Crouter et al. predicted EE using a heart-rate monitor with an accelerometer [26]. Altini et al. presented activity-specific oxygen-uptake (VO_2_) estimation models combining an accelerometer and HR monitor [27]. Brage et al. also reported that indirect calorimetry modelled by the combination of HR and movement was more accurately than either model [28]. Their results showed that the combined model outperformed the single models, such as models with accelerometer only or HR only.

**Figure 1:**
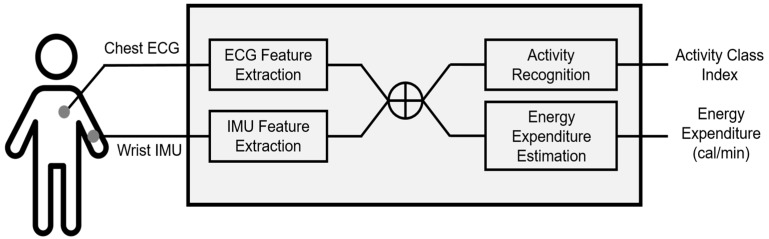
Overall system flow. A cross symbol in a circle indicates the concatenation of two feature vectors.

Based on these findings, we hypothesized that features extracted from ECG and accelerometer signals can be commonly used for both HAR and EE estimation, which enables to develop the integrated healthcare monitoring system with both functions. Therefore, we suggest a novel approach simultaneously taking HAR and EE estimation into considerations based on both accelerometer and ECG. Figure 1 describes the proposed system. Features extracted from ECG and accelerometer signals respectively are concatenated and then input to each module of HAR and EE estimation. The sensor locations in Figure 1 correspond to the database collected in this study and is not limited to these locations in the proposed system.

## 3. Sensors and Database

### 3.1. Wearable Sensors

**Figure 2:**
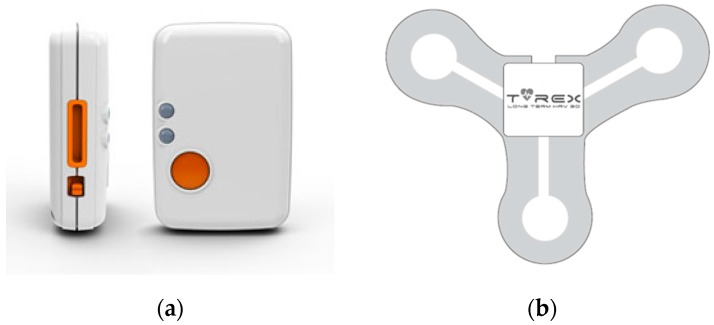
Sensors used in this study: (**a**) Shimmer3. This picture was obtained from its official website (http://www.shimmersensing.com/); (**b**) T-REX TR100A attached on a patch-type electrode. This picture was obtained from its official manual.

The wearable sensors used in this study are shown in Figure 2. We use an inertial measurement unit (IMU) sensor called Shimmer3 (Shimmer Research, Dublin, Ireland) and an ECG sensor called T-Rex TR100A (Taewoong Medical, Gimpo, Korea). Additionally, to obtain the ground-truth energy expenditure, a metabolic gas analysis system with a face mask (Quark CPET, COSMED, Rome, Italy) is also used. Calibration was performed before each test in order to acquire reliable reference energy expenditure data. The calibration procedure consists of gas analyzer and flow/volume calibration. The gas analyzer calibration adjusts the baseline of the CO_2_ sensor and the gain of the O_2_ sensor to ensure the accuracy of the measurement. The flow/volume calibrations are performed by moving the piston in and out for 10 inspiratory and expiratory strokes using a 3-L calibration syringe. After the calibration procedures are complete, the software displays whether the results are within the acceptable range. If one or more values are outside of the acceptable range the calibration must be repeated. Shimmer3 is an accurate high-performance IMU that integrates 10-degree-of-freedom inertial sensing via accelerometer, gyroscope, magnetometer, and altimeter. The sampling rate is set to 128 Hz, which is high enough to capture the details of human daily movements. This sensor offers a strap and a snap clip, providing full mobility, unrestricted movement and comfort for a participant, even during dynamic activities such as running. T-Rex TR100A is a wearable ECG sensor with a disposable patch-type electrode. The sampling rate is set to 256 Hz. This sensor is light and provides comfort for a participant and a patch-type electrode offers good adhesion without the feeling of irritation. Moreover, it maintains stable mounting state when performing dynamic activities. In this study, we use heart-rate variabilities (HRV) calculated from raw signals obtained by the ECG sensor. We acquire 31 HRV parameters from raw ECG signals using Kubios HRV software [29], which is a widely used software for HRV analysis (see Table 1).

### 3.2. Database

**Figure 3:**
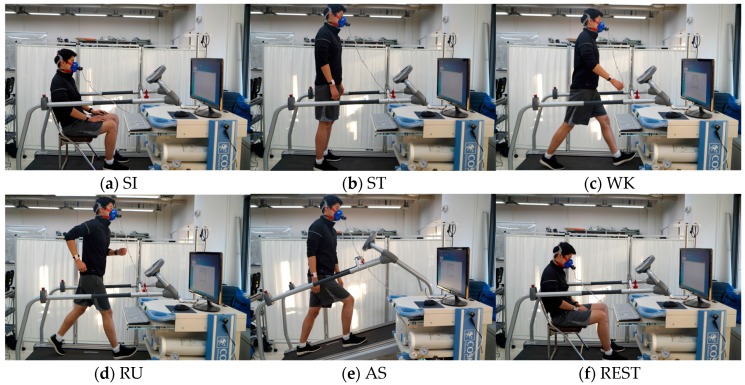
Experiment pictures for all six activities.

Thirteen subjects (9 males and 4 females, mean ± standard deviation age, 25.7 ± 3.1 years) were recruited for this study. Subjects were asked to fast and avoid caffeine-containing beverages and nicotine for 4 h prior to the experimental tasks. They were also asked to abstain from alcohol and vigorous exercise 24 h prior to each experiment [30]. Each participant was required to perform five basic ambulatory activities—sitting (abbreviated as SI), standing (ST), walking (WK), running (RU), ascending (AS)—and rest (REST). During SI and ST activities, no specific hand movements or position guidelines were proposed, and in the case of dynamic activities, the subjects’ natural arm motion was induced. All activities were performed on the treadmill in the experimental room, as shown in Figure 3. Static activities were performed during 5 min, and dynamic activities were performed during 10 min. As also shown in the Figure, a participant was asked to wear IMU sensors on both his/her arms, attach an ECG sensor to his/her chest, and wear a face mask to measure metabolism during a task. Activity recognition was evaluated for these five activities, and energy expenditure estimation is performed for all activities, including rest, i.e., six activities. Activity during rest looks the same as that during SI but needs to be separately considered. Because rest is sitting right after performing a dynamic activity, the physiological states of these two activities are obviously different.

The experimental task was approved by the institutional review board at the Korea Institute of Science and Technology (2016-009).

### 3.3. Preprocessing

During the tasks, participants wore two IMU sensors on both of their arms, but only the data from each individual’s dominant arm were used for further analysis. Raw data from the dominant arm were segmented with a time window of 2 s without overlap. Then, four widely used time- and frequency-domain features were extracted for every time window: RMS, standard deviation, dominant frequency, and energy [31]. The typical window length of accelerometer signal varied from 1 s to 30 s according to the activities to be recognized and the measured attributes. However, the ECG signal usually requires a longer time window, from 30 s to 1 min, to obtain HRV parameters. To align IMU features with ECG features, we selected 1 min as a window length. To do so, we averaged a four-by-30 IMU feature matrix into a four-by-one representative feature vector. HRV parameters were computed using 1 min ECG signal. Therefore, our classification system provided the results of activity recognition and energy-expenditure estimation every minute. To address scale differences among features and subjects, each feature dimension of individual input data was normalized to zero mean and unit variance.

## 4. Activity Recognition

As our first experiment, we examined the activity-recognition performance with respect to the input data compositions using support vector machines with a linear kernel (Linear SVM) and a radial basis function kernel (RBF SVM), k-nearest neighbors (kNN) and linear discriminant analysis (LDA). We set up three recognition scenarios to evaluate performance as follows and applied the four aforementioned classification methods to these scenarios: (1) IMU only: input data are composed of four time- and frequency-domain features extracted from the accelerometer data acquired from IMU sensors. (2) IMU + ECG: input data are composed of four features from IMU sensors (same as (1)) and 31 HRV parameters extracted from the ECG data. (3) IMU + selected ECG: input data are composed of four features from IMU sensors (same as (1)) and selected HRV parameters extracted from the ECG data. The selection criterion is based on the statistical significance (*p*-value) of the training data. We used the Mann-Whitney U test, which is a nonparametric method for data whose probability distribution is not normal, frequently used to assess the differences in HRV parameters.

To evaluate the validity of the recognition performance, we used a leave-one-subject-out cross-validation strategy. We divided the data from 13 subjects into three groups: training, validation, and testing. For each cross-validation fold, there are training data from 11 subjects, validation data from one subject, and testing data from one subject. There is no overlap of data/subject between groups. Validation data were used to select the optimal parameters for some classifiers, such as SVM (box constraint for both kernels, and gamma for the RBF kernel) and kNN (k, the number of nearest neighbors) for every scenario. Additionally, validation data were also used to find the optimal number of ECG features in the Scenario III. We used the LIBSVM toolbox for the classification [32] and Matlab Statistics Toolbox (The MathWorks, Inc., Natick, MA, USA) for the other classification methods.

### 4.1. Scenario I: IMU Only

**Figure 4:**
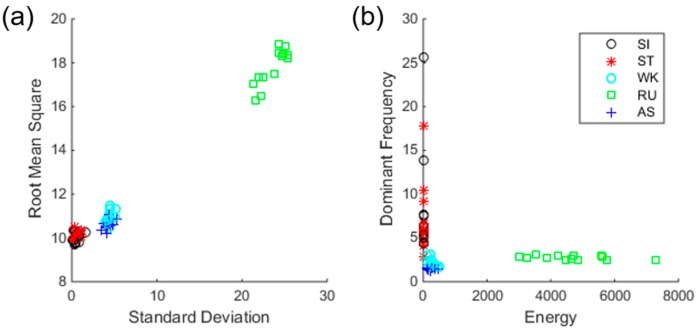
Feature distributions of five activities in (**a**) time and (**b**) frequency domains. Each value represents the average feature value of one subject.

In this recognition scenario, we used four-dimensional input data, and features were extracted from IMU data only. Figure 4 shows average feature values for each subject in time- and frequency-domains, respectively. As shown in the figure, static (SI and ST) and dynamic activities (WK, AS, and RU) are clearly distinguished. However, SI and ST of static activity, WK and AS of dynamic activity are somewhat overlapping, suggesting the difficulty of classification.

Table 2 shows the confusion table for SVM, and the overall recognition accuracy is 83.08%. On the other hand, RBF SVM achieved an accuracy of 76.92%. For kNN, the recognition accuracy is 81.15% with an optimal k of 3, as determined by the validation data. LDA obtained 72.12%. As shown in the confusion table, SI and ST are frequently confused with each other, and WK is confused with AS. This trend has also been observed in the confusion table published in [31]. We can conclude that the information obtained from IMU sensors is insufficient to classify static activities in detail because there is not a big difference between SI and ST in the feature domain. It also shows that there is not a big difference between WK and AS in terms of acceleration.

### 4.2. Scenario II: IMU + ECG

In this recognition scenario, we used four-dimensional input data from IMU data and additional 31-dimensional HRV parameters from ECG data. In other words, the input-data dimension in this scenario is 35. As a result, the recognition accuracy of linear SVM improves to 91.73%, while RBF SVM outperforms it with an accuracy of 92.31%. LDA has the largest improvement with the highest accuracy of 94.81%. kNN improves to 87.50% with an optimal k as 1. The result shows that all methods experience improvement, large or small, due to the additional data. Compared to the previous scenario, the confusion table shows that WK is no longer confused with AS with ECG data, but SI and ST are still confused with each other, although this is slightly improved (see Table 3).

### 4.3. Scenario III: IMU + Selected ECG

**Figure 5:**
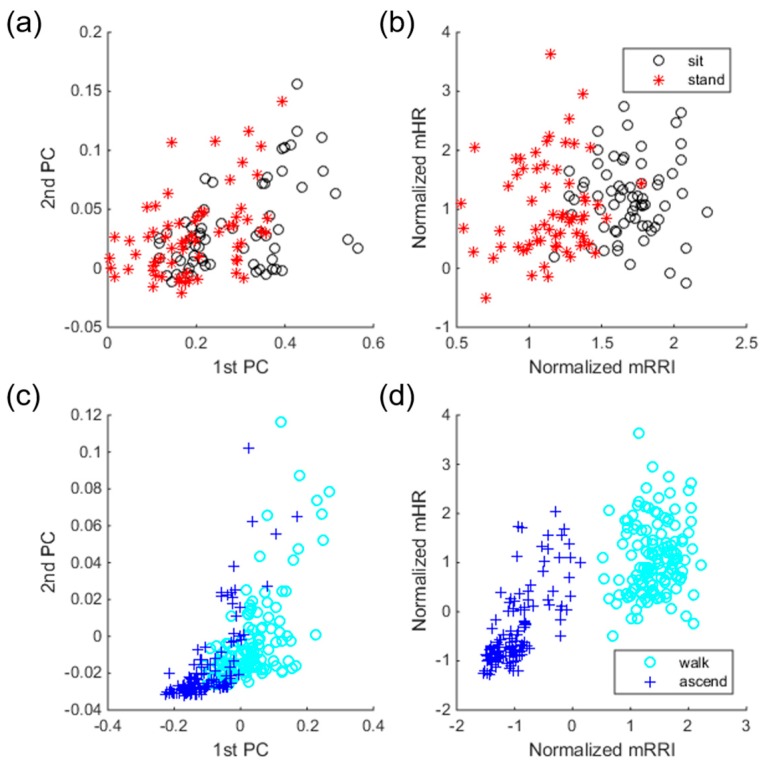
Scatter plots of training samples. (**a**) Samples of classes sitting (SI) and standing (ST) before selection; (**b**) Samples of classed SI and ST after selection; (**c**) Samples of classes walking (WK) and ascending (AS) before selection; (**d**) Samples of classes WK and AS after selection.

We used a statistical test to select better ECG features to solve the drawbacks observed by previous scenarios. The Mann-Whitney U test was used for the evaluation of all univariate differences in HRV parameters between the SI and ST classes, and between the WK and AS classes. To observe the effect of feature selection, we drew two scatter plots before and after feature selection using training samples with their known labels. Samples before feature selection have 31 dimensions and thus cannot be drawn in 2-D or 3-D, so we apply principal component analysis to reduce their dimensions only for the purpose of drawing the plots. Samples after feature selection are represented by two-dimensional vectors because the highest validation accuracy is obtained when two ECG features are used. Specifically, selected features are exactly the same for every 13 folds, mean R-R interval (mRRI) and mean heart rate (mHR). Figure 5 shows the effect of feature selection, separately drawn for two class-pairs, i.e., SI/ST and WK/AS. For samples before selection, the first and second principal components were used for a scatter plot. As shown in the Figure, samples before selection overlap one another, which implies difficulty in classification. However, samples with selected features show better distinctiveness compared to the samples before selection. Moreover, sample distributions before selection in classes SI and ST are more overlapped compared to those in classes WK and AS (see Figure 5a,c). This trend indicates that classification of SI and ST is more difficult than that of WK and AS, which is in line with the confusion table given in Table 3. As shown in the confusion table in Table 4, there are significant improvements in classes SI, ST, and WK, compared to the confusion table in Table 3.

The classification accuracies for all three scenarios are summarized in Table 5. All four methods achieved their highest performances in scenario III, and LDA obtained the highest performance overall, 96.35%.

## 5. Energy-Expenditure Estimation

In the second experiment, we proposed a novel approach to estimate EE during six activities (SI, ST, WK, AS, RU, and REST). As mentioned in Section 3.1., we aim to estimate EE using data obtained from wearable sensors as closely as possible to the EE measured by the metabolic gas analysis system. To estimate the energy consumed during activities, we developed several linear-regression models using multi-sensory input features as independent variables. Generally, a linear-regression model takes the form shown in Equation (1). Assuming a total number of samples *n*, the estimated energy (kcal/min) of the *i*-th sample, $Y_{i}$, is calculated as follows:

(1) $$Y_{i}=\;\beta_{0}+\;\beta_{1}X_{i1}+\;\beta_{2}X_{i2}+\ldots +\;\beta_{k}X_{ik}+\;\epsilon_{i},\;i=1,2,\;\ldots \;,n,\;k\;=\;1,2,\;\ldots ,\;K$$

where $\beta_{k}$ is a *k*-th regression coefficient, $X_{ik}$ represents the *k*-th input feature of the *i*-th sample, and $\epsilon_{i}$ is the error term. The elements of a *K*-dimensional vector β can be simply estimated using an ordinary least-squares method by minimizing the sum of squared error ϵ. The estimate of the regression-coefficient vector $\hat{\beta }$ can be obtained using following closed-form expression (Equation (2)).

(2) $$\hat{\beta }={\left(X^{\prime }X\right)}^{-1}X^{\prime }Y$$

where ’ denotes transpose.

Using this method, we set up four models to compare estimation performances from two perspectives: data and model types. Hereafter, we refer to them as (1) the single model with IMU data only (Model I), (2) the single model with both IMU and ECG data (Model II), (3) the activity-specific model with IMU data only (Model III), and (4) the activity-specific model with both IMU and ECG data (Model IV). For all models, anthropometric features (weight and height) and accelerometer features from IMU data (RMS, standard deviation, dominant frequency, and energy) are commonly used for the initial regression variables. Model II and IV additionally use physiological features (31 HRV parameters).

By comparison of the four models, we expected effects due to (1) the addition of physiological features in EE (data type) and (2) the methods of model construction (single or activity-specific; model type). The effect of (1) can be seen by comparing Models I and II and comparing Models III and IV. The effect of (2) can be seen by comparing Models I and III and comparing Models II and IV.

Among multi-sensory features, the selection criterion of regression variables is based on the statistical significance (*p*-value < 0.05) in the regression model generated by training data. To evaluate each model’s estimation performance, we used the root-mean-square error (RMSE) between the EE values (kcal/min) predicted by a model and the values actually observed by the metabolic gas analysis system.

To validate the generated regression model, we also used a leave-one-subject-out cross-validation strategy. Data from 12 subjects were used to select the optimal regression variables and compute their coefficients. Data from the remaining subject were used to test the generated model. The performance reported afterwards is the average EE or the average RMSE after 13-fold cross-validation. The average RMSE values for each activity are reported in Table 6.

To investigate each effect on estimation performance, a two-way ANOVA was conducted to compare the main effects of types of data and model and the interaction effect between type of data and model on the EE estimation performance. Data type included two levels (IMU and IMU + ECG) and model type consisted of two levels (single and activity-specific).

### 5.1. Effect of Data Type

**Figure 6:**
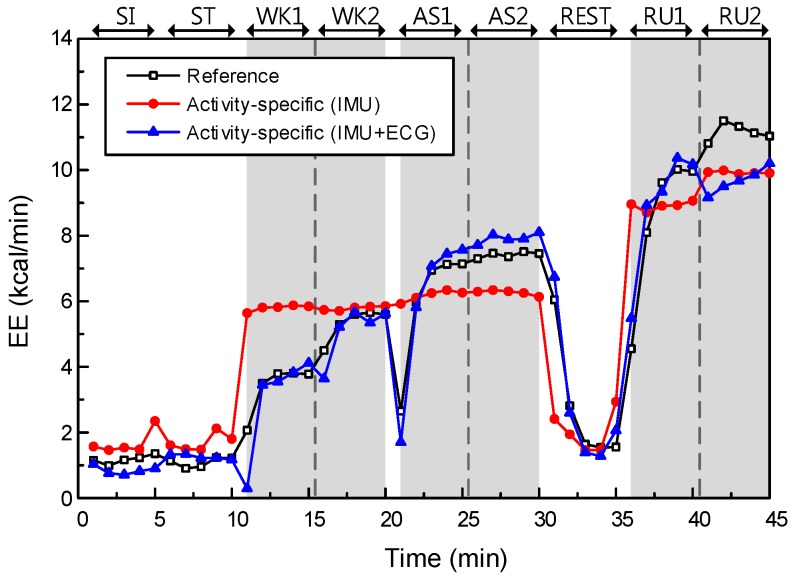
The effect of data type on EE estimation performance, in the case of the activity-specific model (subject 10). Gray-shaded regions indicate dynamic activities (WK, AS, and RU).

First, we investigated the effect of data type on EE performance. The models without physiological features are Models I and III, while the models with additional physiological features are Models II and IV. As mentioned above, the initial features of Models I and III are two anthropometric features and four accelerometer features, and they are used to construct least-squares fits of their models to the training data. Models II and IV used additional physiological features from ECG data, i.e., a 37-dimensional feature vector in total, to construct a least-squares fit. The EE estimation performances of Models III and IV are described in Figure 6.

The main effect of data type yielded an F ratio of F(1, 308) = 38.69, *p* < 0.001, indicating a significant difference in RMSEs between the models with IMU only (Models I and III; μ = 1.59, σ = 0.68) and models with IMU + ECG (Models II and IV; μ = 1.03, σ = 0.38). This result shows that the models with additional physiological features could significantly improve the estimation performance, relative to the models without physiological features.

### 5.2. Effect of Model Type

Next, we compared two types of models in EE estimation. In Models I and III, one single model is generated by the training data for all six activities. In Models II and IV, unlike in the single model, we generated two regression models for each activity type: static and dynamic. Static activities include SI, ST and REST, while dynamic activities are WA, AS and RU.

**Figure 7:**
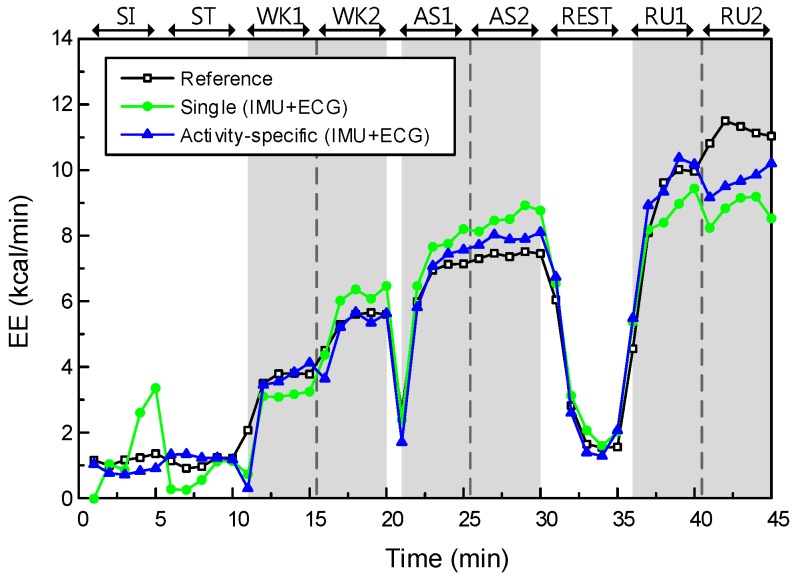
The effect of data type on EE estimation performance, in the case of the activity-specific model (subject 10). Gray-shaded regions indicate dynamic activities (WK, AS, and running (RU)).

The main effect of model type yielded an F ratio of F(1, 308) = 8.58, *p* < 0.005, indicating a significant difference in RMSEs between the single models (Models I and II; μ = 1.44, σ = 0.58) and the activity-specific models (Models III and IV; μ = 1.18, σ = 0.64). This result shows that the activity-specific model could improve the estimation performance significantly, relative to the single model. The EE estimation performance of Models II and IV are described in Figure 7.

We confirmed that both effects are statistically significant on the EE estimation performance. In other words, the addition of physiological features and the use of an activity-specific model have significant impacts on the performance improvement. By comparing these two effects, we can see that data type has a greater impact on the EE estimation performance than the effect of model type. However, there was no significant interaction effect between data and model types (*p*-value = 0.90).

Consequently, Model IV generates 26 regression models computed from each cross validation fold (13-fold) and each activity (static and dynamic). Representatively, Table 7 shows final regression models computed from 1st cross-validation fold.

## 6. Discussion

As mentioned earlier, HAR and EE estimation are important information provided by the mobile healthcare system. The HAR problem has been investigated for a long time by many researchers, and the reported performances of existing approaches seem to be sufficiently high, even for commercialization. The reason we tried to propose a novel approach for this widely known problem is that it is crucial to understand different characteristics of static and dynamic activities for accurate HAR and EE estimation in our daily lives. The accelerometer, as is also well known, has been a good tool for HAR and EE estimation, but we found significant drawbacks, such as the confusion of some activities (SI/ST and WK/AS) and in estimating energy expenditures for activities involving little or no movement (but obviously consuming energy). In this study, we found a significant role of human physiological signals (specifically, HRV parameters) for both problems and an impact of the activity-specific model on EE estimation.

### 6.1. Activity Recognition

Our findings show that selected ECG features indeed improved classification performance dramatically, especially for some labels that were confused when we used IMU features only. Moreover, the selected ECG features are the same for all 13 folds. In other words, the power of the selected ECG features is verified with different training data. Therefore, it is necessary to further consider the details of these two selected features: mRRI and mHR.

By definition, mRRI is the average interval of two successive R peaks. Differences between the mRRI values for the SI and ST classes are shown to be statistically significant by the Mann-Whitney U test (for the SI class, μ = 0.86, σ = 0.12; for the ST class, μ = 0.77, σ = 0.10, and *p*-value = 5.51 × 10^−6^). This means that the interval between R-R peaks is longer for a sitting activity than for a standing activity, and the longer RR interval indicates slower heartbeat due to that activity. We can observe a statistically significant difference in mRRI values between the WK and AS classes (for the WK class, μ = 0.61, σ = 0.08; for the AS class, μ = 0.47, σ = 0.07, *p*-value = 3.15 × 10^−6^). In the same way, the ascending activity makes the heart beat faster than walking.

Moreover, we can clearly interpret these trends with mHR, the mean heart rate. The mHR values also show such differences for both pairs of activity classes (for the SI class, μ = 71.23, σ = 9.11; for the ST class, μ = 79.89, σ = 10.29, *p*-value = 0; for the WK class, μ = 100.82, σ = 12.58; for the AS class, μ = 130.64, σ = 18.32, *p*-value = 0). 

Then, the question is whether ECG features alone can classify activity classes. We additionally developed new scenarios with ECG-only features: (4) ECG-only, and (5) selected ECG-only, and tested these scenarios with LDA which yielded the highest performance in previous scenarios. As a result, we obtained 68.65% (σ = 6.74) with scenario IV, and 69.04% (σ = 7.11). Specifically, predicted labels in scenario V showed that there is no misclassification at all between the SI and ST classes, and between the WK and AS classes, as we statistically investigated above. However, low classification performance of these new scenarios was due to the confusion between static activity classes (SI and ST) and WK classes.

According to these findings, our physiological signals, especially some of HRV parameters associated with the information of the heartbeat, can help to recognize more accurately some activities for which movements are not easily recognizable with accelerometer signals only. In conclusion, the characteristics of the ECG and the IMU are complementary in terms of HAR, so that using these features together can improve performance than using each of them.

### 6.2. Energy Expenditure Estimation

In EE estimation, we generated four models with respect to the data and model types. The effects of data and model types on EE estimation performance were statistically significant in terms of RMSE with reference data from the metabolic gas analysis system. Moreover, the effect of data type was greater than the effect of model type. However, the effect of model type was a dramatic improvement, in static activities specifically. Comparing the RMSEs for static and dynamic activities separately, the difference between Models II and IV was statistically significant for static activities only (*p*-value = 9.71 × 10^−7^, t(1, 38) = 5.83, for Model II; μ = 1.02, σ = 0.54, for Model IV; μ = 0.55, σ = 0.26). This result indicates that the proposed model has made a significant improvement in EE estimation for activities involving little or no physical movement, as expected.

We found that the generated regression models have common variables. Common variables in Models II and IV indicate significant contributions of some features, among all 37 features we used (two anthropometric, four accelerometer, and 31 HRV features). Surprisingly, mHR, which had a great impact on activity recognition as well, was selected by all 13 folds. In other words, the additional information about heart rate is also useful in EE estimation. The crucial role of mHR in both problems means a lot in terms of system construction. By simply computing the average heart rate, we can improve EE estimation performance for static and dynamic activities that reflect daily life.

### 6.3. Limitations and Future Work

We proposed a novel approach to recognize human ambulatory activities and estimate EE using our database composed of IMU and ECG signals, collected from 13 subjects. We have successfully demonstrated that using additional ECG signals, especially adding specific HRV parameters, has resulted in performance improvements for both issues. However, there is obvious limitation that our database has been collected under controlled laboratory environment with subject at specific ages. For broader application of the proposed system, it should be verified with different subject groups such as age, gender, race etc. Because the characteristic of physiological signals providing useful information on heart is highly dependent on these effects. Therefore, system performance may also be enhanced with more sample numbers of the same group with the testing subject.

Considering daily life monitoring using our approach, our database may be limited to a few activities classes. However, we set up the models not for every single activity class but for each activity category (static or dynamic), even though there are other activities beyond six activity classes that we used, our system is expected to yield reasonable performance by applying either static or dynamic model. Moreover, to adapt continuously collected daily life data, system should be updated regularly with new training data. In other words, how to effectively process the vast amount of data and incorporate them into the system will be a new challenge. Future work can be done to address these issues with active learning or selective sampling to regularly update individual models.

## Figures and Tables

**Table 1:** Summary of heart-rate variability (HRV) parameters [29].

Parameters			Units	Description
Time-Domain	1	mRR	ms	The average of RR intervals
	2	SDRR	ms	Standard deviation of RR intervals
	3	mHR	1/min	The average heart rate
	4	SDHR	1/min	Standard deviation of instantaneous heart rate values
	5	RMSSD	ms	Square root of the mean squared differences between successive RR intervals
	6	NN50	count	Number of successive RR interval pairs that differ more than 50 ms
	7	pNN50	%	NN50 divided by the total number of RR intervals
Frequency-Domain	8	VLF	Hz	Peak in very low frequency range (0 to 0.04 Hz)
	9	LF	Hz	Peak in low frequency range (0.04 to 0.15 Hz)
	10	HF	Hz	Peak in high frequency range (0.15 to 0.4Hz)
	11	pVLF	ms^2^	Absoulte powers of VLF bands
	12	pLF	ms^2^	Absoulte powers of LF bands
	13	pHF	ms^2^	Absoulte powers of HF bands
	14	prcVLF	%	Relative powers of VLF bands = VLF(ms^2^)/total power(ms^2^) × 100%
	15	prcLF	%	Relative powers of LF bands = LF(ms^2^)/total power(ms^2^) × 100%
	16	powHF	%	Relative powers of HF bands = HF(ms^2^)/total power(ms^2^) × 100%
	17	nLF	n.u.	Powers of LF bands in normalized units = LF(ms^2^)/(LF + HF)(ms^2^)
	18	nHF	n.u.	Powers of HF bands in normalized units = HF(ms^2^)/(LF + HF)(ms^2^)
	19	LF/HF	-	Ratio between LF and HF band powers
Nonlinear-Domain	20	SD1	ms	Standard deviations of the Poincaré plot (short-term variability)
	21	SD2	ms	Standard deviations of the Poincaré plot (long-term variability)
	22	ApEn	-	Approximate entropy
	23	SampEn	-	Sample entropy
	24	D_2_	-	Correlation dimension
	25	Alpha1	-	Short-term fluctuations of detrended fluctuation analysis (DFA)
	26	Alpha2	-	Long-term fluctuations of detrended fluctuation analysis (DFA)
	27	Lmean	beats	Mean line length of diagonal lines in recurrence plot (RP)
	28	Lmax	beats	Maximum line length of diagonal lines in RP
	29	REC	%	Recurrence rate (percentage of recurrence points in RP)
	30	DET	%	Determinism (percentage of recurrence points which form diagonal lines in RP)
	31	ShanEn	-	Shannon entropy of diagonal line lengths’ probability distribution

**Table 2:** Confusion table for Linear SVM in scenario I.

		Predicted Label					Total	RC
		SI	ST	WK	RU	AS		
Ground Truth	SI	33	31	1	0	0	65	50.77
	ST	21	44	0	0	0	65	67.69
	WK	1	0	98	0	31	130	75.38
	RU	0	0	0	130	0	130	100
	AS	0	0	3	0	127	130	97.69
Total		55	75	102	130	158	520	
PR		60.00	58.67	96.08	100	80.38		

The abbreviations are: SI = sitting, ST = standing, WK = walking, RU = running, AS = ascending, RC = Recall, PR = Precision.

**Table 3:** Confusion table for Linear support vector machine (SVM) in scenario II.

		Predicted Label					Total	RC
		SI	ST	WK	RU	AS		
Ground Truth	SI	49	13	3	0	0	65	75.38
	ST	9	56	0	0	0	65	86.15
	WK	0	2	128	0	0	130	98.46
	RU	0	0	0	130	0	130	100
	AS	0	0	0	0	130	130	100
Total		58	71	131	130	130	520	
PR		84.48	78.87	97.71	100	100		

**Table 4:** Confusion table for Linear SVM in scenario III.

		Predicted Label					Total	RC
		SI	ST	WK	RU	AS		
Ground Truth	SI	53	7	5	0	0	65	81.54
	ST	6	59	0	0	0	65	90.77
	WK	0	0	130	0	0	130	100
	RU	0	0	0	130	0	130	100
	AS	0	0	1	0	129	130	99.23
Total		59	66	136	130	129	520	
PR		89.83	89.39	95.59	100	100		

**Table 5:** Performances in recognition scenarios I, II, and III.

Classification Methods	Scenario I	Scenario II	Scenario III
Linear SVM	83.08 (7.51)	91.73 (7.03)	95.77 (3.73)
RBF SVM	76.92 (6.05)	92.31 (5.63)	95.96 (3.15)
kNN	81.15 (12.44)	87.50 (3.82)	94.04 (5.16)
LDA	72.12 (11.08)	94.81 (5.15)	96.35 (4.16)

Numbers in parentheses indicate standard deviations.

**Table 6:** Root-mean-square errors (RMSEs) of four energy-expenditure (EE) estimation models for each experimental task.

EE Estimation Models	Static Activities			Dynamic Activities			Average
	SI	ST	REST	WK	AS	RU	
(1) Single (IMU)	1.11 (0.54)	0.94 (0.37)	1.74 (0.33)	1.48 (0.72)	2.37 (0.81)	2.63 (0.91)	1.71 (0.68)
(2) Single (IMU + ECG)	0.98 (0.55)	0.90 (0.52)	1.18 (0.56)	0.98 (0.39)	1.22 (0.70)	1.74 (1.22)	1.17 (0.31)
(3) Activity-specific (IMU)	0.62 (0.40)	0.48 (0.29)	1.75 (0.44)	1.78 (0.41)	1.92 (0.63)	2.22 (0.93)	1.46 (0.73)
(4) Activity-specific (IMU + ECG)	0.44 (0.18)	0.46 (0.19)	0.76 (0.26)	1.05 (0.29)	1.10 (0.54)	1.54 (1.16)	0.89 (0.42)

Numbers in parentheses indicate standard deviations.

**Table 7:** Final activity-specific regression models in the 1st fold.

EE Estimation Model (IMU + ECG; cal/min)	R^2^
(1) Static activity: 3.32 − 0.020 × Height + 0.061 × Weight + 0.052 × power + 1.68 × mHR + 0.025 × LF + 1.01 × Lmean − 0.48 × REC	0.93
(2) Dynamic activity: −1.38 − 0.030 × Height + 0.14 × Weight − 0.45 × f_dominant + 0.00068 × power − 0.52 × σ + 0.49 × RMS + 2.98 × SDRR + 1.87 × mHR + 108.80 × SDHR + 0.87 × pNN50 + 0.36 × pLF − 110 × SD1 − 3.24 × SD2 − 0.23 × D_2_ − 0.17 × Alpha1	

mHR = average heart rate; LF = peak in low frequency range; Lmean = Mean line length of diagonal lines in recurrence plot; REC = Recurrence rate; f_dominant = dominant frequency; σ = standard deviation of acceleration; RMS = root mean square of acceleration; SDRR = standard deviation of R-R interval; SDHR = standard deviation of heart rate; pNN50 = NN50 divided by the total number of RR intervals (NN50 = Number of successive RR interval pairs that differ more than 50 ms); pLF = Absolute powers in low frequency range; SD1 = Standard deviations of the Poincaré plot (short-term variability); SD2 = Standard deviations of the Poincaré plot (long-term variability); D_2_ = Correlation dimension; Alpha1 = Short-term fluctuations of detrended fluctuation analysis.
